# ZnO nanotherapeutics for the treatment of burn wounds: recent advances: Correspondence

**DOI:** 10.1097/MS9.0000000000000638

**Published:** 2023-04-14

**Authors:** Hadia Hemmami, Deepak Chandran, Ilham Ben Amor, Soumeia Zeghoud, Pran Mohankumar, Talha Bin Emran

**Affiliations:** a Department of Process Engineering and Petrochemical, Faculty of Technology, University of El Oued, El Oued, Algeria; b Renewable Energy Development unit in Arid Zones (UDERZA), University of El Oued, El Oued, Algeria; c Department of Veterinary Sciences and Animal Husbandry, Amrita School of Agricultural Sciences, Amrita Vishwa Vidyapeetham University; d School of Agricultural Sciences, Karunya Institute of Technology and Sciences, Coimbatore, Tamil Nadu, India; e Department of Pharmacy, BGC Trust University Bangladesh, Chittagong, Bangladesh; f Department of Pharmacy, Faculty of Allied Health Sciences, Daffodil International University, Dhaka, Bangladesh

*Dear Editor*,

The biggest organ in the body and the most active immune system organ is the skin, which also serves as the primary barrier between the outside environment and the body’s interior organs^1^. Abrasions, burns, and trauma are the most common causes of acute skin wounds. Burn injuries are the fourth most common type of traumatic injury, frequently rupturing the skin layers and/or subcutaneous tissue and even harming the viscera^2^. It is unfortunate for their family and society when survivors of severe burn injuries pass away or are left with scars, impairments, or abnormalities^3^.

We now have a thorough grasp of how burn wounds develop. Bacterial infection, excessive inflammatory response, and low levels of different growth factors are the key variables influencing how burn wounds progress. The most important complicating condition among these is bacterial infection. Therapeutic approaches including anti-infection, stem cell treatment^4^, and administration of growth factors (GFs) to speed up wound healing^5^ have been employed to treat these wounds in line with the aetiology of burn injuries. These treatments significantly lower the risk of infections and accelerate healing. As the wounded skin is vulnerable to microorganisms, anti-infection is one of these treatments that is particularly important for wound healing. Reactive oxygen species (ROS) or ROS producers, antibiotics, metallic ions and metal oxides^6^,^7^, reactive oxygen species^8^, and other anti-bacterial treatments have all been heavily employed to remove germs from burn wound surfaces. Transdermal or systemic delivery, however, may not guarantee that sufficient medicines are delivered to the infection site. In addition, prolonged antibiotic use may lead to a rise in antibiotic resistance. Another treatment strategy for accelerating healing is the application of growth factors (GFs) to the burn site’s surface. Burn wounds have been treated with a variety of GFs, including keratinocyte formation growth factor, transforming growth factor beta, epidermal growth factor, nerve growth factor, basic fibroblast growth factor, vascular endothelial growth factor, and platelet-derived growth factor^9^. Nonetheless, the therapeutic use of GFs for this purpose is constrained by certain of their physicochemical characteristics, such as poor stability.

During the past 20 years, nanotechnology has rapidly advanced, opening up possibilities for the treatment of several ailments. Drugs, biomacromolecules (such as DNA, peptides, and proteins), therapeutic materials (such as certain metals/metal oxides, chitosan), and medicines with at least one dimension of nanoscale structure are all considered nanotherapeutics^10^. When used to treat bacterial infections, nanotherapeutics have a number of benefits, including the ability to: (1) enhance bacterial medication interactions or change bacterial drug metabolism to enhance bacterial drug effects; (2) increase drug concentration at infection sites, which reduces drug dosage and lessens toxic side effects; (3) To combat bacterial resistance, improve medication penetration across tissue barriers and bacterial biofilms; and (4) improve the stability and prolo polymeric^8^, Metal oxide, metal, and other nanotherapeutics have been used extensively to treat burn wounds because of the benefits listed above. Metal nanomaterials (Ag, Au, ZnO, and Cu) show broad-spectrum anti-bacterial action by dissolving bacterial biofilms, eradicating bacterial DNA, or producing ROS to thwart bacterial development^11^. The toxicity of these nanotherapeutics should be taken into account, though, since it may limit future in-vivo use. Polymeric nanomaterials (such as polysaccharide, polyester, and polyamide) have better biocompatibility and biodegradability compared to metal nanoparticles, and they have been widely employed in a variety of biomedical sectors. Due to the positive charge of the polymer, certain cationic polymeric nanoparticles, like chitosan, exhibit bactericidal and bacteriological capabilities. They stick to bacterial surfaces and cause damage to the membrane wall, which inhibits microbial growth^12^. Another key tactic for avoiding wound infection involves encasing antibiotics in polymeric nanomaterials , and encasing GFs in such materials to accelerate wound healing has also been well studied^13^. In conclusion, nanotherapeutics have been produced to treat burn wounds and show good anti-bacterial action, decreased bacterial medication resistance, and shorter wound healing time. They provide a potential alternative for the therapeutic treatment of burns^14^.

As they offer a wide range of anti-bacterial properties^15^–^17^, Burn wounds as well as various other cutaneous infections have been successfully treated with metal and metal oxide nanotherapeutics (for instance, Gold, Ag, and ZnO NPs)^18^. These nanotherapeutics can also overcome bacterial resistance in a number of additional ways, including suppression of biofilm formation, oxidative stress, DNA damage, disruption of enzyme function, cell wall disintegration, and plasmid damage^8^.

A ZnO NP Zinc, a micronutrient that is required for tissue regeneration and can boost the number of keratinocytes to quicken wound healing, has a long half-life in live cells^19^. The fundamental anti-bacterial process is the trapping of ZnO NPs by the bacterial cell wall, which renders the bacteria’s cell membrane ineffective and cleaves it^20^. Moreover, ZnO NPs can improve cell adhesion, proliferation, and migration via GF-mediated pathways since Zn possesses semiconductor properties. ZnO NPs may potentially function as permanent sources of ionic Zn for wound therapy because of their antibacterial, anti-inflammatory, and low cytotoxicity properties^21^, and they have been successfully used to treat a variety of burn wounds.

For the manufacture of bandages, ZnO NPs are often put on polysaccharide or polymer, which makes administration simple. Animal models have been used extensively to create bandages with ZnO NPs for treating different kinds of burn burns. For instance, a keratin–chitosan bandage that had been loaded with ZnO NPs showed significant porosity, which was encouraging for the stimulation of fibroblasts^22^. This bandage showed high tensile strength, biodegradation, and anti-bacterial activity. In-vivo tests showed that this bandage might speed up collagen and skin cell growth, which would aid in the healing of wounds. In contrast, plant extracts like Barleria gibsoni have also been employed to stabilise ZnO NPs; these NPs likewise shown strong anti-bacterial activities and were successfully used as antimicrobial formulations to treat burn infections in rats^23^.

## Ethical approval

Nil.

## Consent

None declared.

## Source of funding

This compilation is a correspondence article written by its authors and required no substantial funding to be stated.

## Author contributions

H.H.: conceptualization, data curation, writing—original draft preparation, writing—reviewing and editing. Deepak Chandran: data curation, writing—original draft preparation, writing—reviewing and editing. I.B.A., S.Z.: writing—original draft preparation, writing—reviewing and editing. P.M., T.B.E.: writing—reviewing and editing, visualisation, supervision.

## Conflicts of interest disclosure

All authors declare that there exist no commercial or financial relationships that could, in any way, lead to a potential conflict of interest.

## Provenance and peer review

Not commissioned, externally peer-reviewed.

## Acknowledgement

All the authors acknowledge and thank their respective Institutes and Universities.
